# Supplementary material for: Bidirectional regulation of postmitotic H3K27me3 distributions underlie cerebellar granule neuron maturation dynamics
Source: eLife. 2023 Apr 24;12:e86273. doi: 10.7554/eLife.86273 (PMC10181825; doi:10.7554/eLife.86273)
Supplement: Figure 1—source data 2. [file elife-86273-fig1-data2.zip › Figure 1 - source data 1/Figure 1 - source data 1 - Uncropped Blots Annotated.pptx]

## Slide 1
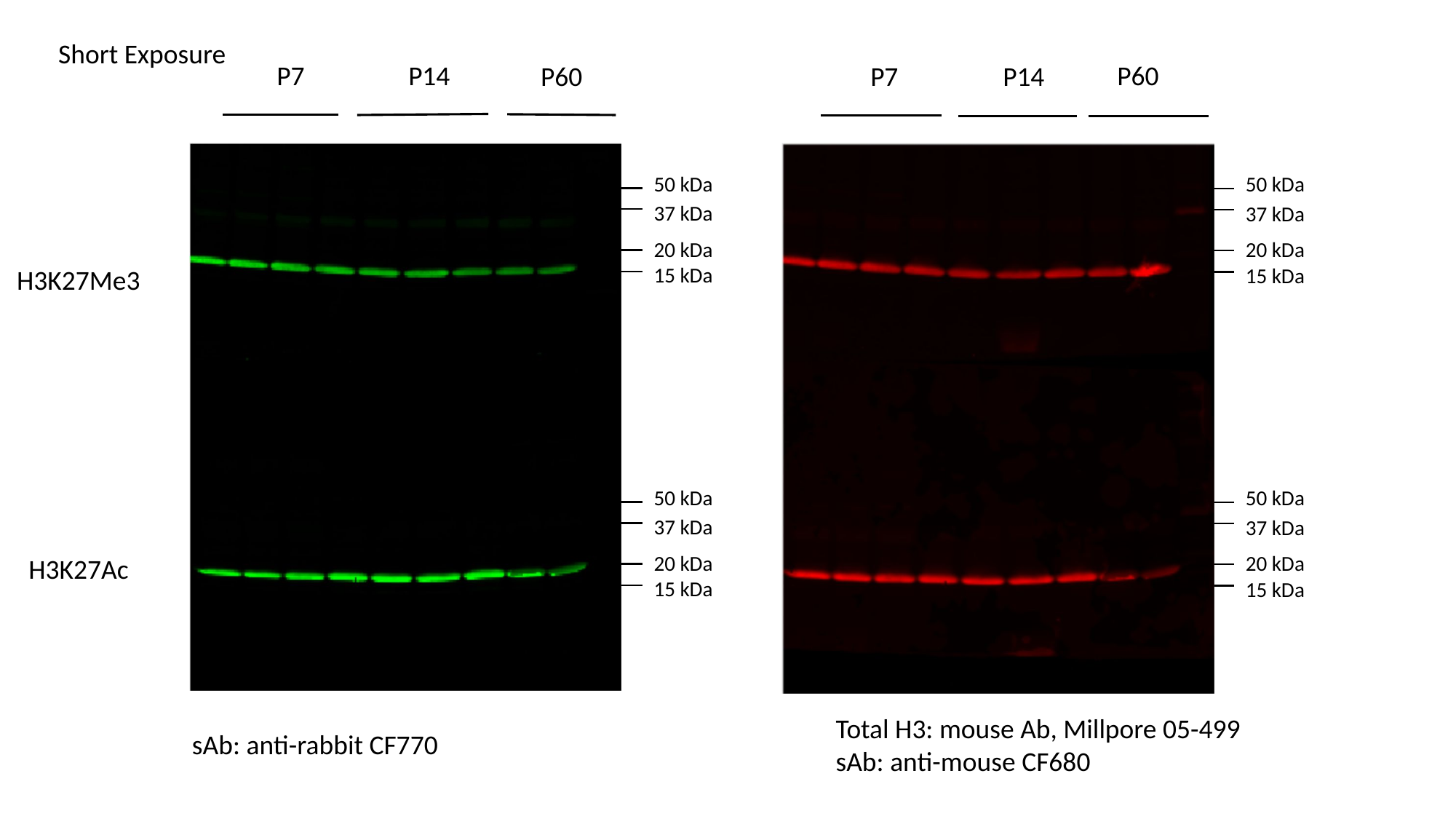

Short Exposure
P7
P14
P60
P60
P14
P7
50 kDa
50 kDa
37 kDa
37 kDa
20 kDa
20 kDa
15 kDa
15 kDa
H3K27Me3
50 kDa
50 kDa
37 kDa
37 kDa
20 kDa
20 kDa
H3K27Ac
15 kDa
15 kDa
Total H3: mouse Ab, Millpore 05-499
sAb: anti-mouse CF680
sAb: anti-rabbit CF770
