# Supplementary material for: Bidirectional regulation of postmitotic H3K27me3 distributions underlie cerebellar granule neuron maturation dynamics
Source: eLife. 2023 Apr 24;12:e86273. doi: 10.7554/eLife.86273 (PMC10181825; doi:10.7554/eLife.86273)
Supplement: Figure 1—figure supplement 1—source data 2. [file elife-86273-fig1-figsupp1-data2.zip › Figure S1 - source data 2/Figure S1 - source data 2 - Uncropped Blots Annotated.pptx]

## Slide 1
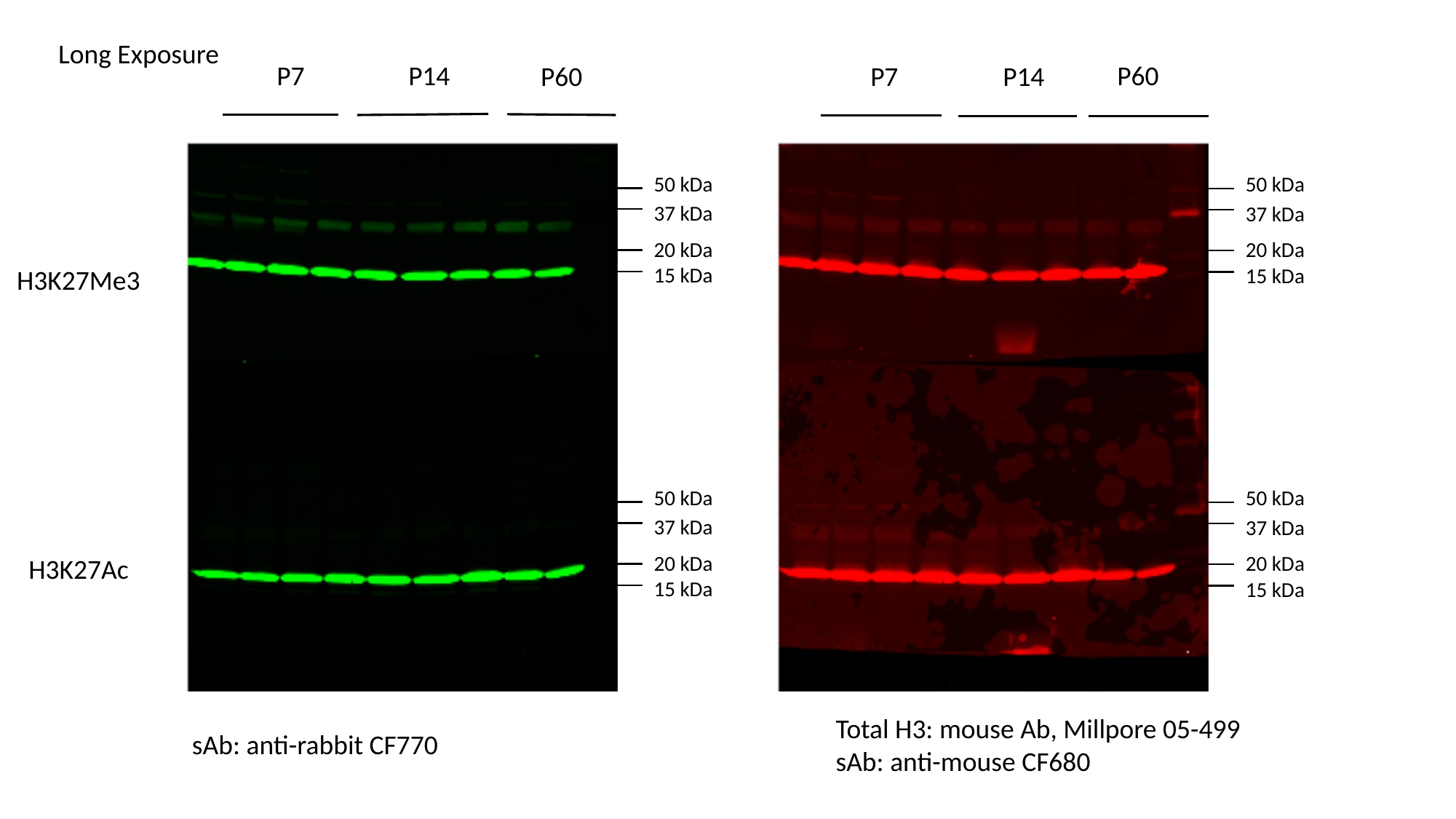

Long Exposure
P7
P14
P60
P60
P14
P7
50 kDa
50 kDa
37 kDa
37 kDa
20 kDa
20 kDa
15 kDa
15 kDa
H3K27Me3
50 kDa
50 kDa
37 kDa
37 kDa
20 kDa
20 kDa
H3K27Ac
15 kDa
15 kDa
Total H3: mouse Ab, Millpore 05-499
sAb: anti-mouse CF680
sAb: anti-rabbit CF770

## Slide 2
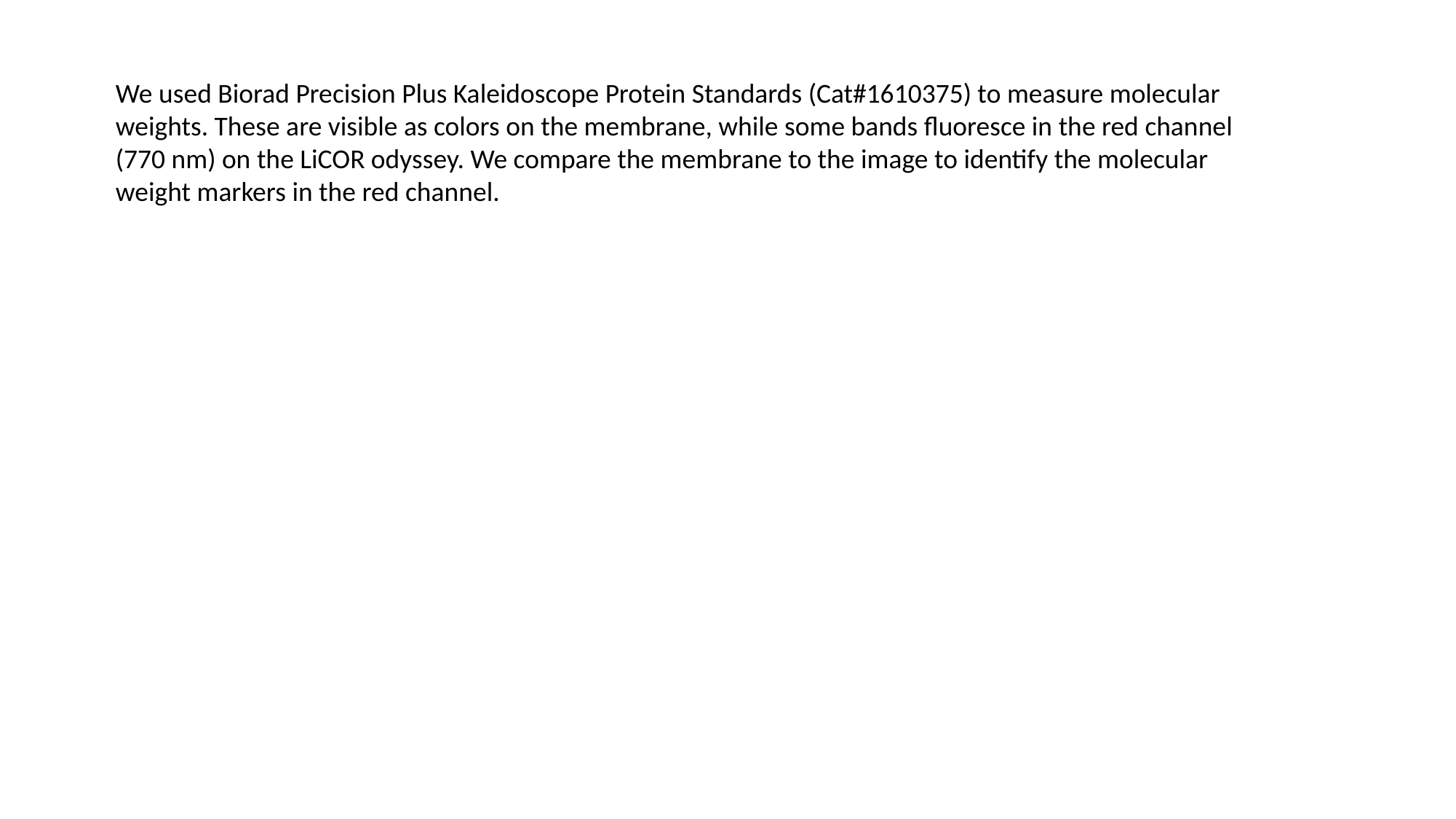

We used Biorad Precision Plus Kaleidoscope Protein Standards (Cat#1610375) to measure molecular weights. These are visible as colors on the membrane, while some bands fluoresce in the red channel (770 nm) on the LiCOR odyssey. We compare the membrane to the image to identify the molecular weight markers in the red channel.
