# Supplementary figures and images for: Bidirectional regulation of postmitotic H3K27me3 distributions underlie cerebellar granule neuron maturation dynamics
Source: eLife. 2023 Apr 24;12:e86273. doi: 10.7554/eLife.86273 (PMC10181825; doi:10.7554/eLife.86273)

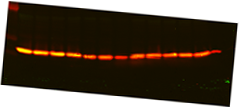

Supplement: Figure 7—source data 1. [file elife-86273-fig7-data1.zip › Figure 7 - source data 1/GSK-126-700-800.tif]

## Slide 1
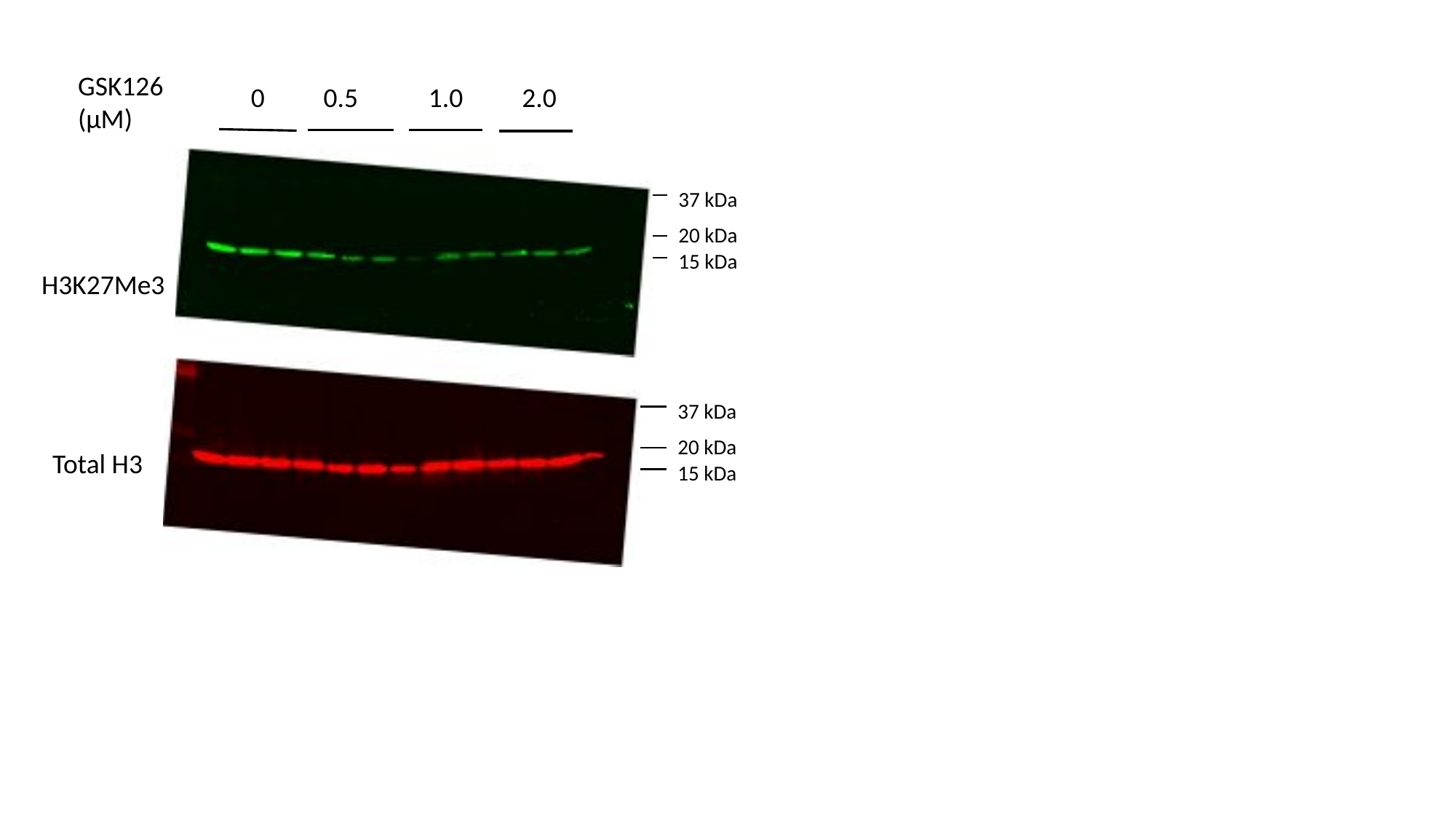

GSK126
(µM)
0
0.5
1.0
2.0
37 kDa
20 kDa
15 kDa
H3K27Me3
37 kDa
20 kDa
Total H3
15 kDa

Supplement: Figure 7—source data 1. [file elife-86273-fig7-data1.zip › Figure 7 - source data 1/Figure 7 - source data 1 - Uncropped Blots Annotated.pptx]

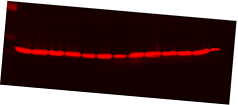

Supplement: Figure 7—source data 1. [file elife-86273-fig7-data1.zip › Figure 7 - source data 1/GSK-126-700.tif]

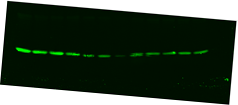

Supplement: Figure 7—source data 1. [file elife-86273-fig7-data1.zip › Figure 7 - source data 1/GSK-126-800.tif]

## Slide 1
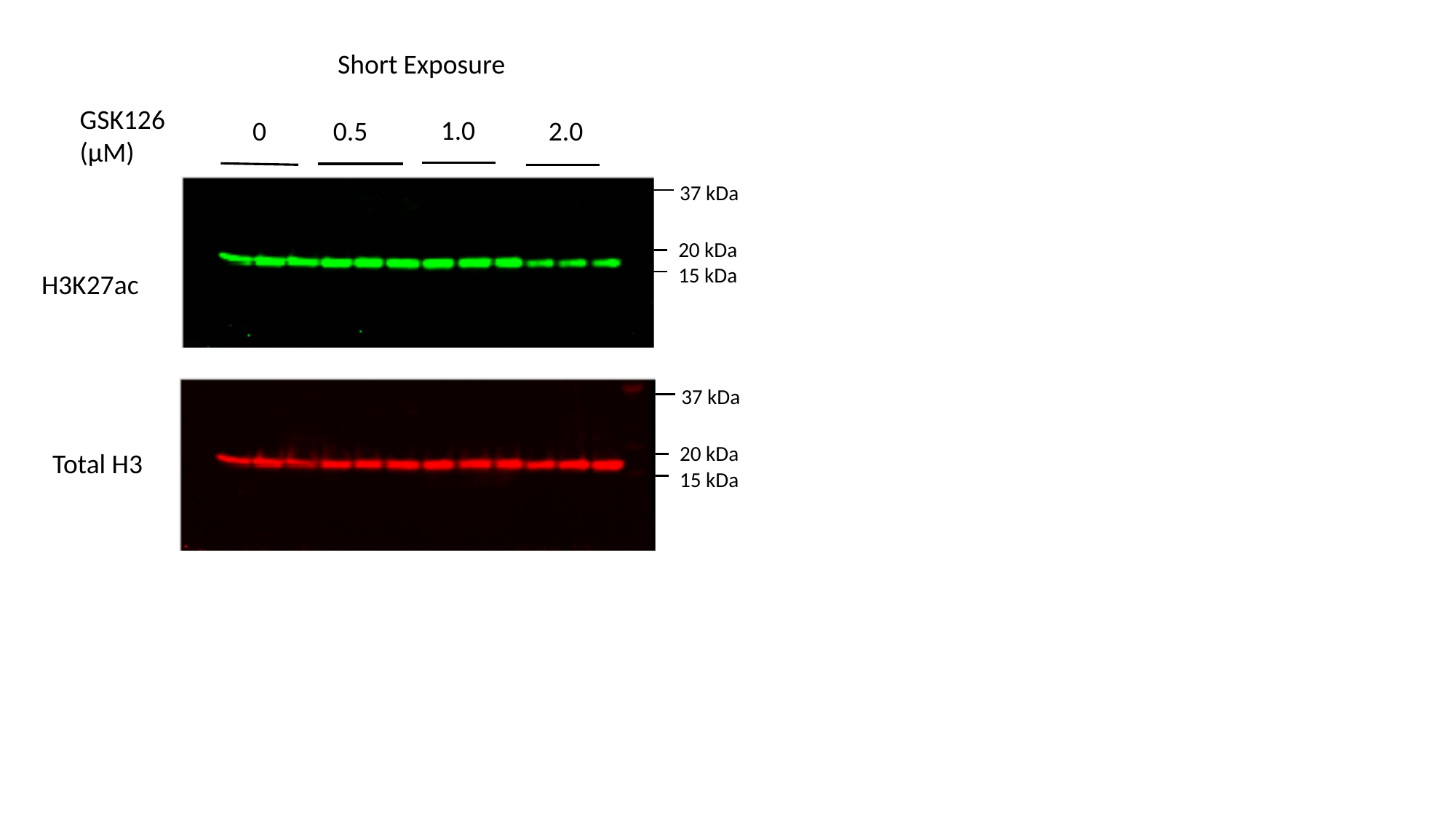

Short Exposure
GSK126
(µM)
1.0
0
0.5
2.0
37 kDa
20 kDa
15 kDa
H3K27ac
37 kDa
20 kDa
Total H3
15 kDa

Supplement: Figure 7—source data 2. [file elife-86273-fig7-data2.zip › Figure 7 - source data 2/Figure 7 - source data 2 - Uncropped Blots Annotated.pptx]

## Slide 1
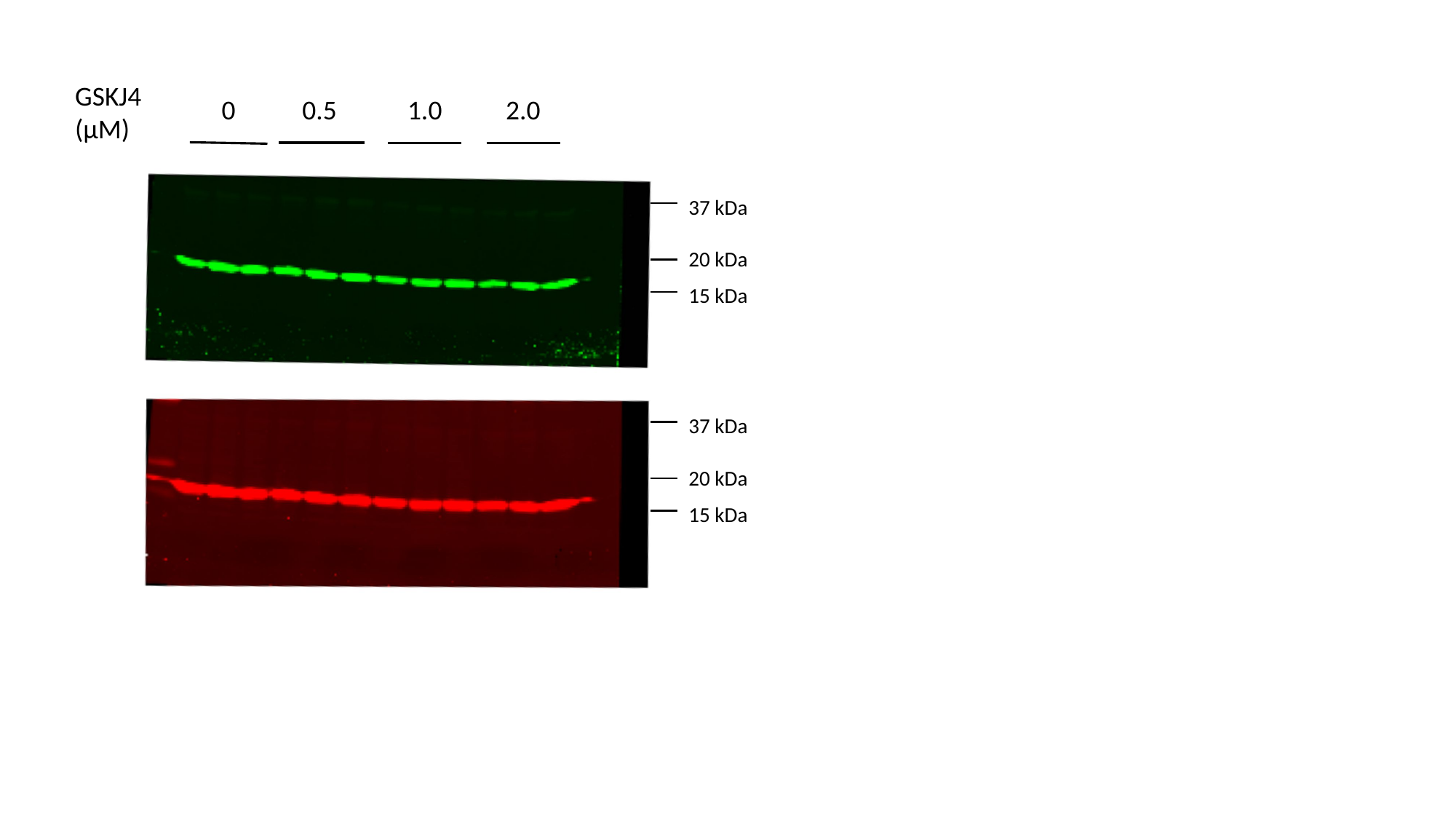

GSKJ4
(µM)
0
0.5
1.0
2.0
37 kDa
20 kDa
15 kDa
37 kDa
20 kDa
15 kDa

Supplement: Figure 7—figure supplement 1—source data 1. [file elife-86273-fig7-figsupp1-data1.zip › Figure S9 - source data 1/Figure S9 - source data 1 - Uncropped Blots Annotated.pptx]

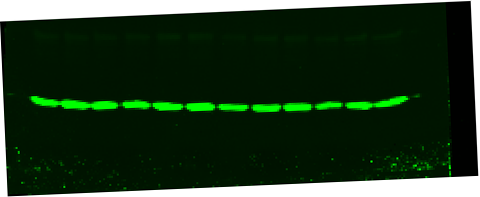

Supplement: Figure 7—figure supplement 1—source data 1. [file elife-86273-fig7-figsupp1-data1.zip › Figure S9 - source data 1/GSK-J4-800d.tif]

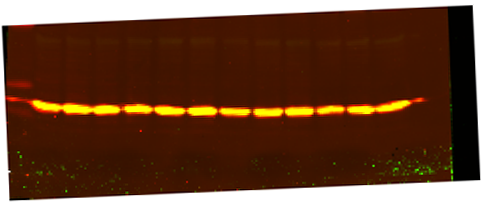

Supplement: Figure 7—figure supplement 1—source data 1. [file elife-86273-fig7-figsupp1-data1.zip › Figure S9 - source data 1/GSK-J4-700-800d.tif]

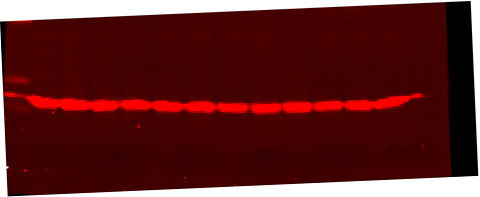

Supplement: Figure 7—figure supplement 1—source data 1. [file elife-86273-fig7-figsupp1-data1.zip › Figure S9 - source data 1/GSK-J4-700d.tif]

## Slide 1
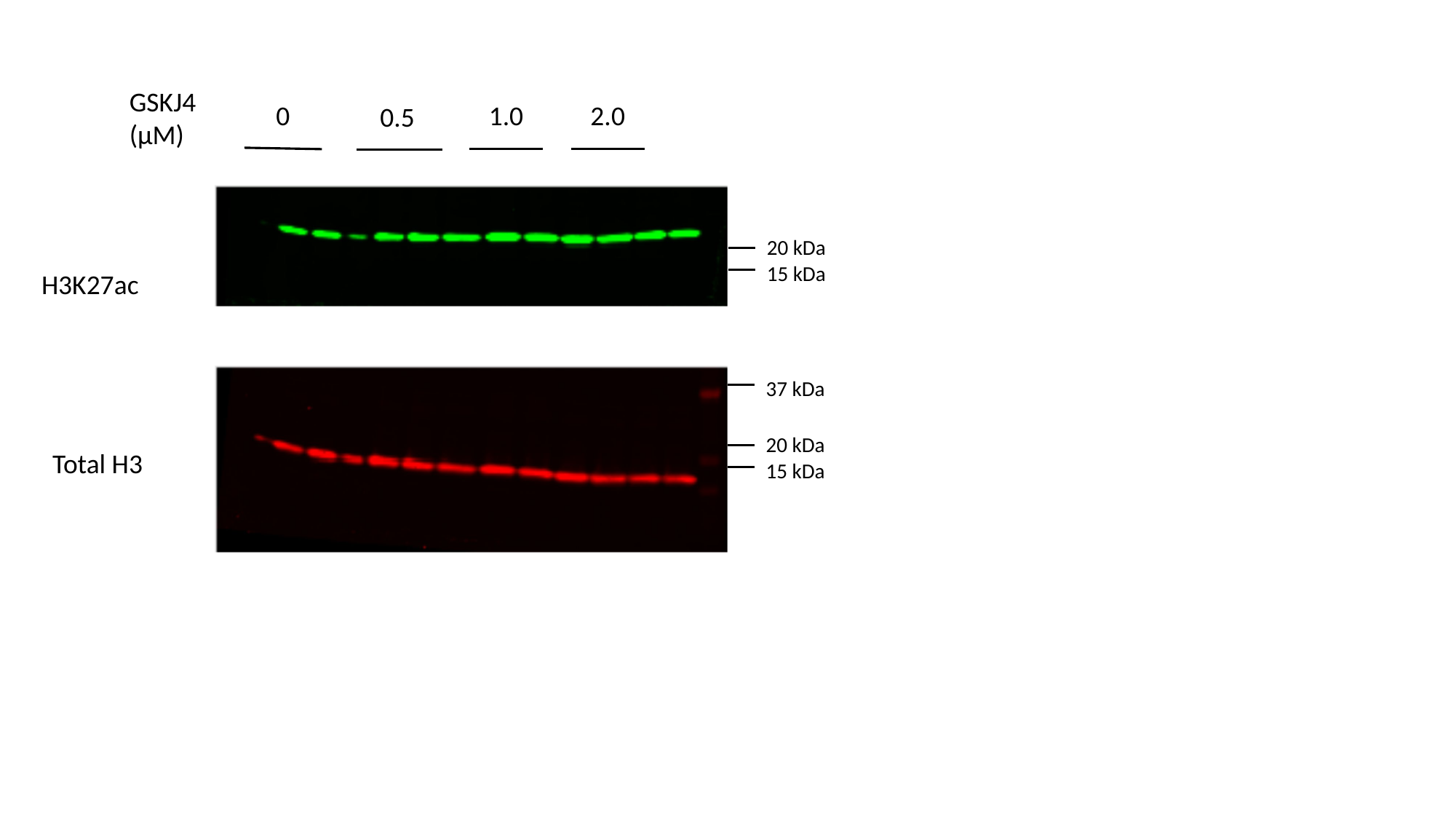

GSKJ4
(µM)
0
1.0
2.0
0.5
20 kDa
15 kDa
H3K27ac
37 kDa
20 kDa
Total H3
15 kDa

Supplement: Figure 7—figure supplement 1—source data 2. [file elife-86273-fig7-figsupp1-data2.zip › Figure S9 - source data 2/Figure S8 - source data 2 - Uncropped Blots Annotated.pptx]
